# Supplementary figures and images for: Arabidopsis thaliana exudates induce growth and proteomic changes in Gluconacetobacter diazotrophicus
Source: PeerJ. 2020 Jul 28;8:e9600. doi: 10.7717/peerj.9600 (PMC7676354; doi:10.7717/peerj.9600)

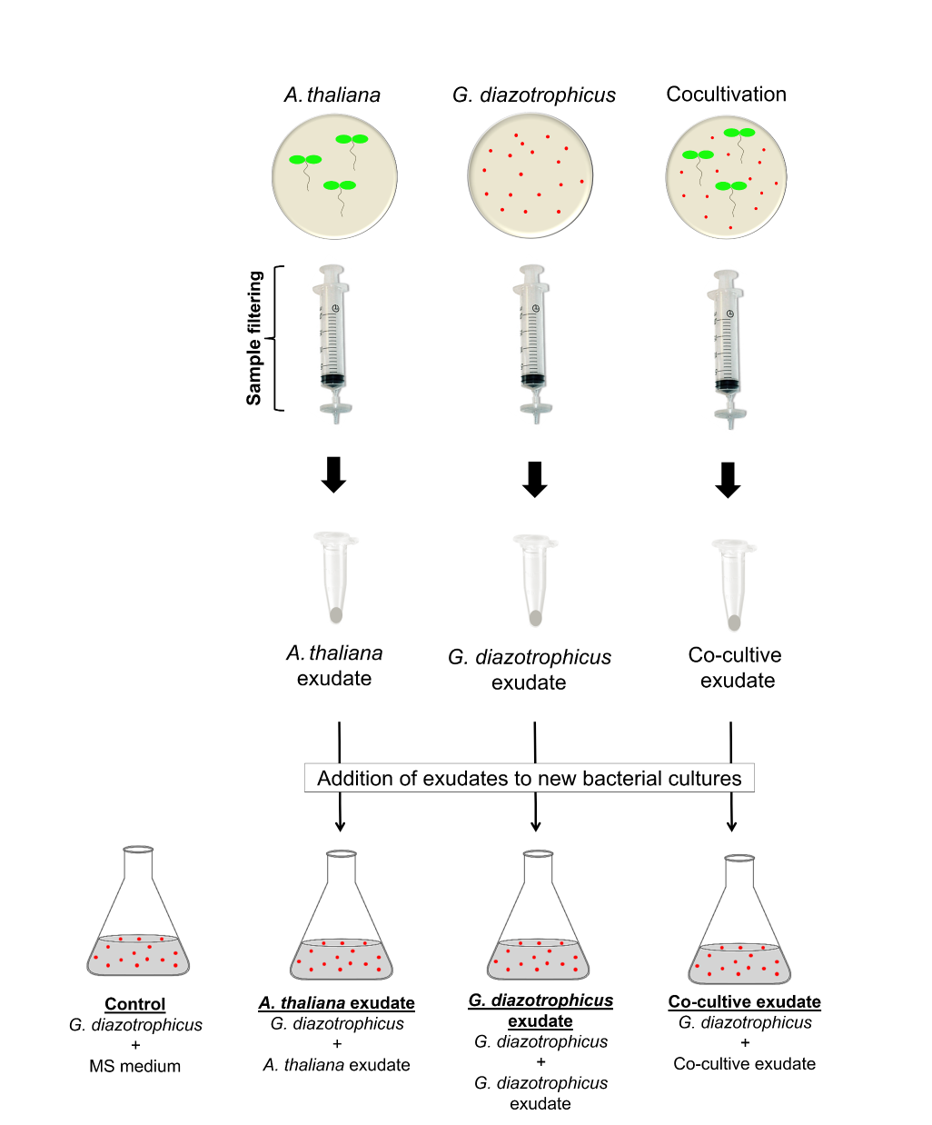

Supplement: Supplemental Information 1 — Exudates were obtained from three different sources: non-inoculated A. thaliana seedlings (A. thaliana exudate), G. diazotrophicus cultures (G. diazotrophicus exudate), and G. diazotrophicus/A. thaliana cocultivation (Co-cultive exudate). Afterward, the obtained exudates were added in the new bacterial cultures. [file peerj-08-9600-s001.png]

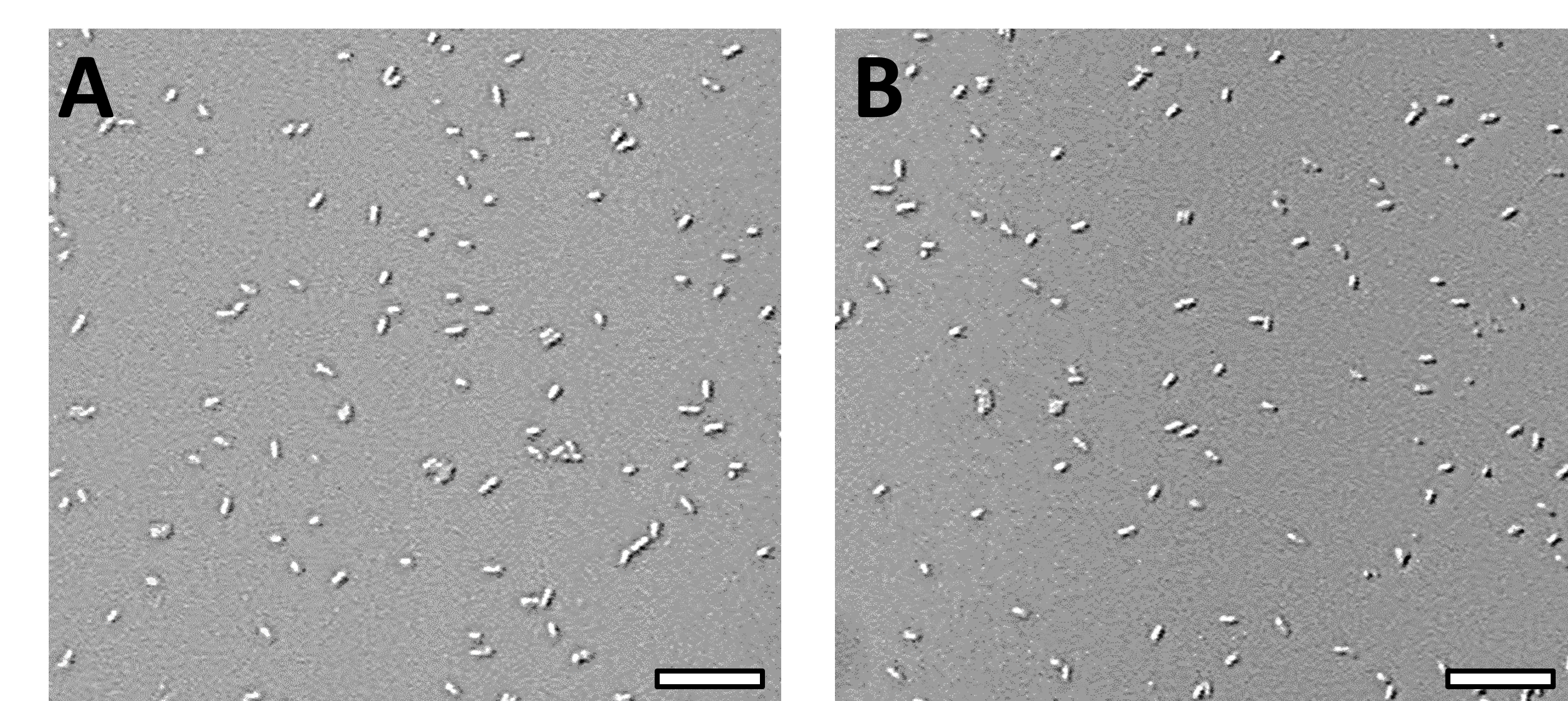

Supplement: Supplemental Information 2 — G. diazotrophicus cells were inoculated in half-strength MS medium without A. thaliana seedlings (A) and in half-strength MS medium with A. thaliana seedlings (B), and, after 24 h, its cells morphology was analyzed through optical microscopy. [file peerj-08-9600-s002.png]

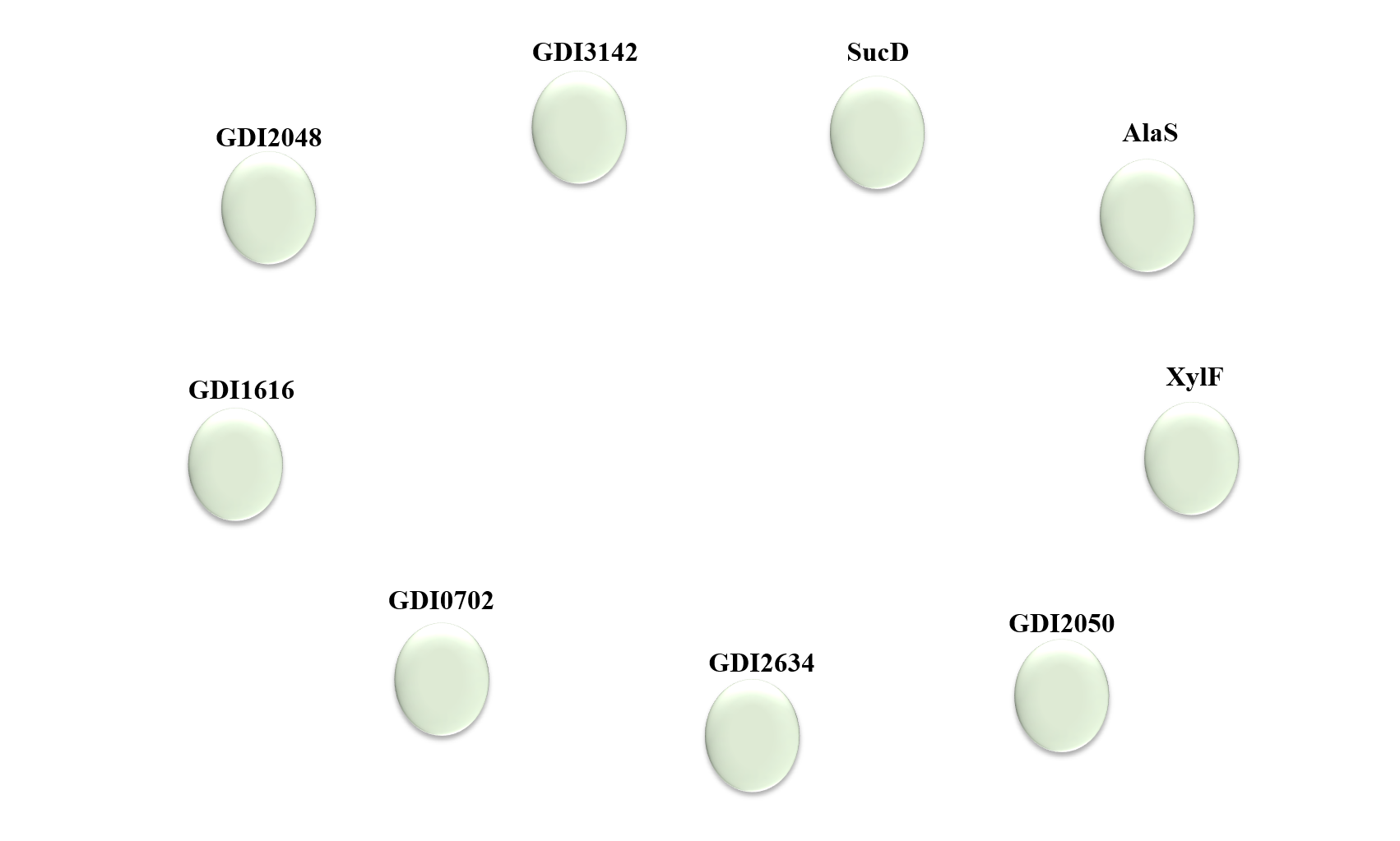

Supplement: Supplemental Information 3 [file peerj-08-9600-s003.png]
