# Supplementary material for: Arabidopsis thaliana exudates induce growth and proteomic changes in Gluconacetobacter diazotrophicus
Source: PeerJ. 2020 Jul 28;8:e9600. doi: 10.7717/peerj.9600 (PMC7676354; doi:10.7717/peerj.9600)
Supplement: Supplemental Information 4 [file peerj-08-9600-s004.docx]

| **Table S1. Proteins identified in *G. diazotrophicus* co-cultivated with *A. thaliana* seedlings** | | | | | | | | | | |  |
| --- | --- | --- | --- | --- | --- | --- | --- | --- | --- | --- | --- |
| **Description** | **Max Score** | **Accession** | **Reported Peptides** | **TIC Control1** | **TIC Control2** | **TIC Control3** | **TIC Cocultive1** | **TIC Cocultive2** | **TIC Cocultive3** | **T Test** | **Fold Change** |
| Putative thioredoxin protein | 3216,44 | A9HSA5 | 4 | 33751 | 30658 | 24268 | 37284 | 50837 | 45318 | 0,02 | 1,50 |
| 50S ribosomal protein L23 | 8093,88 | RL23 | 6 | 139687 | 131733 | 81037 | 169607 | 214910 | 150527 | 0,04 | 1,52 |
| S-(hydroxymethyl)glutathione dehydrogenase | 1057,50 | A9HIP1 | 4 | 20030 | 29121 | 23621 | 39279 | 35962 | 35730 | 0,01 | 1,52 |
| Glucose-6-phosphate 1-dehydrogenase | 5629,12 | A9H0G0 | 27 | 103430 | 103637 | 109496 | 164855 | 171322 | 150346 | 0,00 | 1,54 |
| 30S ribosomal protein S2 | 11963,12 | RS2 | 16 | 145855 | 162126 | 201375 | 246785 | 287118 | 263141 | 0,01 | 1,56 |
| Oxidoreductase domain protein | 6904,23 | A9HDU1 | 14 | 96699 | 86520 | 88299 | 134062 | 150014 | 157743 | 0,00 | 1,63 |
| Protein TolR | 1454,51 | A9HAZ8 | 3 | 17609 | 27104 | 28875 | 46987 | 29408 | 43945 | 0,04 | 1,64 |
| Putative transcriptional Regulator, MarR family | 6080,51 | A9HJY0 | 4 | 78448 | 77952 | 72891 | 121449 | 140808 | 119568 | 0,01 | 1,67 |
| Adenylyl-sulfate kinase | 4170,78 | A9H0W3 | 21 | 49644 | 53251 | 63662 | 103585 | 81955 | 93644 | 0,01 | 1,68 |
| Glycine--tRNA ligase beta subunit | 1189,10 | A9HM86 | 11 | 15017 | 14252 | 16064 | 30672 | 23946 | 22012 | 0,03 | 1,69 |
| Signal recognition particle protein | 2042,15 | A9HS68 | 12 | 29441 | 24941 | 34073 | 47852 | 62335 | 44235 | 0,02 | 1,75 |
| Putative Squalene--hopene cyclase | 350,53 | A9HGY2 | 2 | 10423 | 12796 | 16207 | 25729 | 23981 | 19901 | 0,01 | 1,77 |
| Uncharacterized protein | 769,19 | A9HJS1 | 4 | 16233 | 24033 | 19039 | 41545 | 32276 | 30970 | 0,01 | 1,77 |
| 30S ribosomal protein S8 | 4884,06 | RS8 | 6 | 40983 | 56085 | 52886 | 99327 | 104670 | 67306 | 0,03 | 1,81 |
| Histidine--tRNA ligase | 728,77 | SYH | 6 | 17239 | 15922 | 19311 | 31650 | 35415 | 28424 | 0,00 | 1,82 |
| 6-phosphogluconate dehydrogenase | 20323,17 | A9H324 | 16 | 206490 | 201421 | 227186 | 389591 | 384713 | 411606 | 0,00 | 1,87 |
| Argininosuccinate synthase | 4334,52 | ASSY | 10 | 47604 | 48757 | 77560 | 116747 | 81851 | 126581 | 0,02 | 1,87 |
| Putative metallopeptidase | 3042,59 | A9HRE6 | 19 | 57605 | 49931 | 60178 | 104745 | 126733 | 101394 | 0,01 | 1,98 |
| 50S ribosomal protein L6 | 8968,96 | RL6 | 9 | 100504 | 141566 | 110728 | 198537 | 313664 | 203194 | 0,04 | 2,03 |
| Phosphoglycerate mutase | 4303,62 | A9H397 | 11 | 54425 | 55289 | 60939 | 132555 | 99988 | 114086 | 0,01 | 2,03 |
| Dihydrolipoyl dehydrogenase | 1282,86 | A9HJB6 | 8 | 35286 | 29728 | 27015 | 68110 | 77359 | 47192 | 0,03 | 2,09 |
| Glycine dehydrogenase (decarboxylating) | 1964,67 | A9HM48 | 20 | 31255 | 32070 | 30820 | 78349 | 53647 | 69424 | 0,02 | 2,14 |
| Glutamine synthetase | 15678,34 | A9H7Z5 | 23 | 142897 | 156448 | 150399 | 299111 | 349136 | 339121 | 0,00 | 2,20 |
| Orotate phosphoribosyltransferase | 3799,75 | A9HII0 | 5 | 21302 | 27185 | 33254 | 57053 | 80297 | 49686 | 0,02 | 2,29 |
| Glutamate--cysteine ligase | 1745,48 | A9H108 | 7 | 20233 | 20632 | 16606 | 36041 | 50926 | 45398 | 0,01 | 2,30 |
| 30S ribosomal protein S1 | 11955,40 | A9H459 | 30 | 124656 | 116459 | 141975 | 312926 | 317139 | 280187 | 0,00 | 2,38 |
| 50S ribosomal protein L14 | 2991,02 | RL14 | 4 | 35846 | 61657 | 47033 | 111224 | 138476 | 94010 | 0,01 | 2,38 |
| Glucose-6-phosphate 1-dehydrogenase | 837,57 | A9H326 | 5 | 13672 | 9440 | 7282 | 26139 | 34610 | 16104 | 0,04 | 2,53 |
| 50S ribosomal protein L5 | 1646,31 | A9H3M8 | 4 | 24126 | 20713 | 18220 | 70814 | 59954 | 32190 | 0,05 | 2,58 |
| ATP-binding subunit ClpX | 3303,25 | CLPX | 11 | 22460 | 28007 | 28501 | 79673 | 78050 | 50943 | 0,02 | 2,64 |
| Flavodoxin | 1571,93 | ISPG | 6 | 15664 | 14456 | 18308 | 40359 | 69158 | 35947 | 0,04 | 3,00 |
| Conservev protein | 1962,62 | A9GZU8 | 7 | 22135 | 15534 | 23859 | 64219 | 69227 | 53338 | 0,00 | 3,04 |
| Biotin carboxylase protein | 4423,06 | A9HEX0 | 13 | 17624 | 25964 | 32133 | 99028 | 98256 | 69205 | 0,01 | 3,52 |
| Elongation factor G | 10183,21 | A9HS02 | 31 | 52378 | 49494 | 61013 | 217005 | 275051 | 148193 | 0,02 | 3,93 |
| TonB-dependent receptor | 2074,37 | A9H932 | 14 | 22953 | 23750 | 18618 | 101893 | 90411 | 75760 | 0,00 | 4,10 |
| Ketol-acid reductoisomerase (NADP(+)) | 2463,85 | ILVC | 5 | 34296 | 27772 | 14132 | 108051 | 114221 | 98585 | 0,00 | 4,21 |
| ABCF-EttA | 506,79 | A9H4G2 | 5 | 7890 | 7804 | 8980 | 41279 | 44986 | 18219 | 0,04 | 4,23 |
| Aconitate hydratase | 10076,64 | A9HEZ2 | 44 | 35900 | 39113 | 51106 | 384691 | 513512 | 172286 | 0,04 | 8,49 |
| MetE | 11719,34 | A9HNX4 | 38 | 24032 | 25433 | 27299 | 302511 | 153657 | 277740 | 0,02 | 9,56 |
| Uncharacterized protein | 10074,54 | A9HB99 | 4 | 239019 | 213334 | 197292 | 114853 | 58842 | 114840 | 0,00 | 0,44 |
| Porin | 2838,53 | A9HPF6 | 9 | 102858 | 83279 | 51665 | 26322 | 40505 | 41768 | 0,04 | 0,46 |
| Putative periplasmic binding proteins | 4390,16 | A9H577 | 8 | 103326 | 117164 | 61770 | 41852 | 49136 | 41619 | 0,05 | 0,47 |
| Alcohol dehydrogenase GroES domain protein | 1094,65 | A9H073 | 4 | 35485 | 38427 | 21563 | 15381 | 15695 | 14945 | 0,04 | 0,48 |
| Succinate--CoA ligase | 2866,13 | A9HRF1 | 5 | 92502 | 77862 | 104364 | 48643 | 34085 | 56833 | 0,01 | 0,51 |
| 10 kDa chaperonin | 4835,59 | A9HPH9 | 5 | 114740 | 111393 | 103259 | 74452 | 38617 | 63537 | 0,02 | 0,54 |
| D-xylose ABC transporter | 13607,54 | A9HNP0 | 18 | 414442 | 428026 | 295712 | 197710 | 257442 | 200834 | 0,02 | 0,58 |
| Cold-shock DNA-binding domain protein | 34042,72 | A9HK34 | 5 | 458365 | 439645 | 340025 | 293334 | 158056 | 283508 | 0,02 | 0,59 |
| Uncharacterized protein | 3273,24 | A9HEI5 | 3 | 48100 | 41192 | 34436 | 27585 | 27184 | 22797 | 0,02 | 0,63 |
| Inosine-guanosine kinase | 1497,69 | A9H9C0 | 4 | 45122 | 46068 | 47903 | 33247 | 27948 | 26722 | 0,00 | 0,63 |
| Alanine--tRNA ligase | 1109,99 | A9HL73 | 13 | 48410 | 41792 | 41805 | 34662 | 17908 | 32540 | 0,04 | 0,64 |
| Extracellular solute-binding protein family 1 | 9768,71 | A9HPE1 | 13 | 343361 | 368298 | 345479 | 249603 | 247211 | 198858 | 0,00 | 0,66 |
| Putative myo-inositol catabolism | 308,57 | A9H599 | 2 | 19542 | 32941 | 3607 | 6838 | 9225 | 7697 | 0,17 | 0,42 |
| Dihydroxy-acid dehydratase | 1431,00 | A9HA40 | 12 | 40648 | 39839 | 166366 | 38655 | 20747 | 51734 | 0,20 | 0,45 |
| 50S ribosomal protein L31 | 19562,45 | RL31 | 5 | 130407 | 163602 | 261511 | 84584 | 34590 | 145954 | 0,07 | 0,48 |
| Ribose-5-phosphate isomerase A | 4440,82 | A9H338 | 7 | 117131 | 43380 | 58250 | 38760 | 32471 | 48191 | 0,14 | 0,55 |
| Electron transport protein SC | 2810,37 | A9H4T2 | 6 | 27950 | 38016 | 52538 | 25595 | 22291 | 26532 | 0,09 | 0,63 |
| Putative chemotaxis protein cheA | 1318,51 | A9HHF0 | 11 | 46204 | 54519 | 65069 | 48856 | 16332 | 39649 | 0,08 | 0,63 |
| Porin | 1072,61 | A9HAM5 | 5 | 25302 | 29601 | 42426 | 19180 | 16293 | 27582 | 0,07 | 0,65 |
| N-carbamoyl-L-amino acid amidohydrolase | 548,58 | A9HJT7 | 2 | 77857 | 74627 | 52645 | 28782 | 51167 | 54671 | 0,05 | 0,66 |
| Peptidase protein | 2526,34 | A9HET1 | 17 | 124101 | 91742 | 61155 | 53750 | 68658 | 59859 | 0,11 | 0,66 |
| Ribulokinase | 2768,19 | A9HPD7 | 12 | 171106 | 138785 | 88571 | 81889 | 94623 | 86234 | 0,10 | 0,66 |
| Putative peroxiredoxin osmC | 4229,53 | A9H0U0 | 3 | 113993 | 139775 | 84690 | 61062 | 90483 | 72050 | 0,06 | 0,66 |
| Phosphoenolpyruvate carboxykinase (ATP) | 1315,99 | A9H8M9 | 13 | 32834 | 36379 | 32869 | 15550 | 19925 | 32623 | 0,08 | 0,67 |
| Geranyltranstransferase | 1389,50 | A9HIR3 | 3 | 65044 | 47308 | 44440 | 41857 | 24646 | 38219 | 0,05 | 0,67 |
| Glucose-6-phosphate 1-dehydrogenase | 7891,91 | A9HFE6 | 26 | 176755 | 178263 | 156326 | 115743 | 92127 | 135829 | 0,01 | 0,67 |
| Carbamoyl-phosphate synthase large chain | 2084,48 | A9H1P7 | 27 | 57518 | 57615 | 81051 | 52500 | 28294 | 51506 | 0,06 | 0,67 |
| Phosphate acyltransferase | 463,72 | PLSX | 2 | 47133 | 43365 | 33894 | 30287 | 22300 | 31539 | 0,03 | 0,68 |
| Carboxymethylenebutenolidase | 1479,33 | A9HK54 | 2 | 20777 | 20996 | 15611 | 13807 | 13681 | 11367 | 0,03 | 0,68 |
| Ribose-phosphate pyrophosphokinase | 2961,53 | A9HBZ3 | 9 | 78132 | 67514 | 78994 | 45995 | 45330 | 60981 | 0,01 | 0,68 |
| 60 kDa chaperonin 2 | 14463,91 | CH602 | 32 | 181619 | 278431 | 318279 | 191702 | 153238 | 182925 | 0,08 | 0,68 |
| Ubiquinol oxidase subunit 2 | 6600,33 | A9HK01 | 10 | 189249 | 240518 | 278541 | 178424 | 114423 | 188880 | 0,05 | 0,68 |
| Acetoin(Diacetyl) reductase | 1178,41 | A9HHS9 | 4 | 58768 | 33234 | 31654 | 26382 | 23686 | 34513 | 0,14 | 0,68 |
| ATP-dependent zinc metalloprotease FtsH | 851,88 | A9HB14 | 11 | 52925 | 51414 | 28602 | 36199 | 23304 | 31823 | 0,11 | 0,69 |
| Fumarate lyase | 493,31 | A9H6Y6 | 2 | 20517 | 20906 | 8472 | 13240 | 6937 | 14290 | 0,17 | 0,69 |
| Polyamine aminopropyltransferase | 3545,62 | A9HM56 | 7 | 63579 | 67812 | 79759 | 62635 | 14623 | 70330 | 0,17 | 0,70 |
| Clp protease proteolytic subunit | 4160,24 | A9HRV4 | 9 | 91944 | 93343 | 127060 | 69412 | 80776 | 69943 | 0,05 | 0,70 |
| Putative oxidoreductase | 1561,34 | A9HM21 | 5 | 31935 | 26627 | 30216 | 16952 | 26127 | 20040 | 0,03 | 0,71 |
| 3-alpha-hydroxysteroid dehydrogenase | 660,24 | A9HP05 | 2 | 48541 | 43465 | 41694 | 38278 | 29777 | 28269 | 0,02 | 0,72 |
| Ribosome-binding ATPase YchF | 1777,67 | A9HC06 | 10 | 44472 | 48725 | 46152 | 34965 | 27275 | 38384 | 0,02 | 0,72 |
| Peptidase protein, modulator of DNA gyrase | 1370,26 | A9HEU1 | 10 | 61433 | 62961 | 42894 | 38717 | 32444 | 50237 | 0,07 | 0,73 |
| Putative pilus assembly protein | 727,45 | A9HHL8 | 5 | 37287 | 31321 | 38045 | 27281 | 23560 | 26626 | 0,01 | 0,73 |
| FMN-dependent NADH-azoreductase | 2467,58 | AZOR | 4 | 39644 | 23973 | 48356 | 24812 | 25676 | 31131 | 0,14 | 0,73 |
| Putative 6-phosphogluconolactonase | 1033,18 | A9HJ42 | 5 | 22342 | 18036 | 38236 | 23269 | 15242 | 19347 | 0,19 | 0,74 |
| Putative FeS assembly protein SufD | 385,36 | A9HRY4 | 2 | 21375 | 24875 | 29764 | 21820 | 11194 | 22949 | 0,11 | 0,74 |
| Acetate kinase | 1503,62 | A9HGX6 | 8 | 28993 | 30626 | 26543 | 23734 | 15747 | 23959 | 0,05 | 0,74 |
| Peptide methionine sulfoxide reductase MsrA | 4328,02 | A9HI15 | 4 | 94445 | 68653 | 52418 | 55059 | 44982 | 59155 | 0,13 | 0,74 |
| Periplasmic serine endoprotease DegP-like | 1069,42 | A9HBK9 | 10 | 31696 | 31841 | 46338 | 26522 | 25832 | 28839 | 0,09 | 0,74 |
| 1-deoxy-D-xylulose 5-phosphate reductoisomerase | 1739,65 | A9HKV5 | 11 | 36294 | 34772 | 38081 | 26387 | 27357 | 27003 | 0,00 | 0,74 |
| Electron transfer flavoprotein subunit beta | 7167,35 | A9HEE6 | 12 | 101488 | 113986 | 151994 | 82590 | 96803 | 92594 | 0,08 | 0,74 |
| O-succinylhomoserine sulfhydrylase | 1367,20 | A9HAQ3 | 5 | 64207 | 59183 | 89583 | 49288 | 48942 | 59975 | 0,09 | 0,74 |
| dCTP deaminase | 4442,71 | A9HFN2 | 6 | 32174 | 39138 | 73962 | 42014 | 31918 | 34197 | 0,22 | 0,74 |
| Gamma-glutamyltranspeptidase | 3481,89 | A9HM18 | 18 | 73623 | 73121 | 63941 | 50642 | 43547 | 62903 | 0,03 | 0,75 |
| Aminotransferase | 607,02 | A9H801 | 4 | 24877 | 22023 | 16069 | 13415 | 17556 | 16007 | 0,08 | 0,75 |
| Succinate--CoA ligase [ADP-forming] subunit beta | 2692,44 | A9HRF0 | 15 | 102939 | 112896 | 82350 | 69130 | 81484 | 72428 | 0,05 | 0,75 |
| Thioredoxin reductase | 1845,80 | A9H0A5 | 3 | 55971 | 53628 | 64985 | 49917 | 40592 | 40145 | 0,02 | 0,75 |
| Acireductone dioxygenase | 2348,02 | MTND | 5 | 26915 | 30694 | 32611 | 21318 | 21659 | 24572 | 0,01 | 0,75 |
| DNA-directed RNA polymerase subunit omega | 6377,02 | RPOZ | 2 | 94230 | 142817 | 212232 | 97091 | 106876 | 132483 | 0,19 | 0,75 |
| Putative aminotransferase protein | 591,17 | A9HJX0 | 3 | 20912 | 20610 | 24254 | 19898 | 10119 | 19378 | 0,11 | 0,75 |
| Pyruvate, phosphate dikinase | 2595,20 | A9HEP2 | 23 | 121561 | 112504 | 101008 | 83395 | 67815 | 100586 | 0,04 | 0,75 |
| D-ribose-binding periplasmic protein | 2025,44 | A9HPK6 | 5 | 34645 | 35677 | 55196 | 39250 | 25025 | 30187 | 0,14 | 0,75 |
| Conserved protein | 4796,64 | A9HIX3 | 8 | 89337 | 93212 | 134151 | 83820 | 68769 | 86546 | 0,10 | 0,76 |
| Glutamate methylesterase | 2383,93 | A9HHG0 | 9 | 56836 | 58539 | 66531 | 48054 | 38988 | 50394 | 0,02 | 0,76 |
| Uncharacterized protein | 3655,27 | A9HRD5 | 8 | 73755 | 73765 | 54400 | 43243 | 49900 | 60080 | 0,06 | 0,76 |
| Putative phosphoserine aminotransferase | 734,26 | A9HLQ7 | 6 | 26569 | 27955 | 32805 | 23332 | 14964 | 28181 | 0,10 | 0,76 |
| Ribose import ATP-binding protein RbsA | 1883,00 | A9HPC3 | 9 | 81816 | 73340 | 49080 | 53281 | 47176 | 55167 | 0,12 | 0,76 |
| Putative membrane protein | 685,32 | A9H806 | 2 | 17820 | 15353 | 16122 | 10197 | 13333 | 14184 | 0,03 | 0,77 |
| Putative threonine dehydratase catabolic | 1071,30 | A9H381 | 9 | 35363 | 33268 | 25725 | 21948 | 27505 | 22779 | 0,06 | 0,77 |
| Tol-Pal system protein TolB | 9168,71 | A9HB04 | 17 | 221509 | 212741 | 176807 | 149251 | 128409 | 190980 | 0,06 | 0,77 |
| Flagellar motor switch protein FliN | 4498,17 | A9HHD1 | 4 | 63413 | 44447 | 74231 | 53392 | 33793 | 53126 | 0,14 | 0,77 |
| Peptidyl-prolyl cis-trans isomerase D | 2478,14 | A9HJ89 | 21 | 60650 | 57646 | 66605 | 53232 | 36691 | 52558 | 0,05 | 0,77 |
| Peptide chain release factor 2 | 2364,26 | A9HF65 | 7 | 38218 | 48852 | 57412 | 34004 | 28205 | 49555 | 0,13 | 0,77 |
| Ubiquinone biosynthesis C-methyltransferase UbiE | 3501,06 | A9HI27 | 9 | 62859 | 46945 | 64294 | 50284 | 18993 | 66515 | 0,23 | 0,78 |
| Peptidase U62 modulator of DNA gyrase | 564,51 | A9HKF2 | 7 | 30349 | 29754 | 21883 | 21907 | 18044 | 24297 | 0,08 | 0,78 |
| Dihydrolipoyl dehydrogenase | 11018,17 | A9HFH1 | 24 | 365307 | 371505 | 300442 | 255307 | 255231 | 304458 | 0,03 | 0,79 |
| Phosphoglucomutase | 3966,23 | A9HSH5 | 20 | 115236 | 114673 | 110625 | 101832 | 68080 | 97662 | 0,07 | 0,79 |
| Glutamyl-tRNA(Gln) amidotransferase subunit A | 2989,28 | A9HRI9 | 15 | 55258 | 54327 | 95325 | 60111 | 32857 | 68073 | 0,22 | 0,79 |
| 3-oxoacyl-[acyl-carrier-protein] synthase 2 | 3715,98 | A9HRD7 | 9 | 98780 | 87807 | 89695 | 71752 | 43477 | 102776 | 0,19 | 0,79 |
| Cysteine synthase | 5520,68 | A9HAE5 | 15 | 79258 | 96504 | 100420 | 78917 | 48081 | 91165 | 0,14 | 0,79 |
| Glycerol kinase | 15683,53 | A9HHY3 | 26 | 322138 | 345411 | 244989 | 248508 | 251364 | 224706 | 0,08 | 0,79 |
| Alcohol dehydrogenase | 7602,50 | A9HNA5 | 10 | 194012 | 170693 | 196081 | 167004 | 122228 | 157469 | 0,04 | 0,80 |
| Phenylalanine--tRNA ligase beta subunit | 1119,71 | A9H165 | 13 | 31757 | 45774 | 43774 | 34550 | 27361 | 34816 | 0,10 | 0,80 |
| Putative autotransporter protein | 747,73 | A9H4L1 | 9 | 63404 | 59442 | 68280 | 54392 | 41691 | 56363 | 0,04 | 0,80 |
| Glutaredoxin | 32036,04 | A9HJG8 | 7 | 223277 | 246375 | 254128 | 206198 | 146041 | 225400 | 0,08 | 0,80 |
| Phosphate-binding protein PstS | 3472,17 | A9H9X2 | 10 | 93725 | 94205 | 123413 | 79636 | 62960 | 106802 | 0,14 | 0,80 |
| LacI transcriptional regulator | 15517,36 | A9HPB9 | 14 | 400155 | 404665 | 349297 | 266749 | 363655 | 301850 | 0,05 | 0,81 |
| Uncharacterized protein | 2532,82 | A9HB09 | 5 | 44999 | 49036 | 61846 | 48566 | 24655 | 52979 | 0,20 | 0,81 |
| Chemoreceptor mcpA | 3933,34 | A9HHE0 | 16 | 93341 | 104680 | 100014 | 86501 | 74995 | 80007 | 0,01 | 0,81 |
| Alcohol dehydrogenase zinc-binding domain protein | 675,51 | A9HE22 | 3 | 22928 | 27363 | 18169 | 10851 | 13728 | 30933 | 0,29 | 0,81 |
| NADH-quinone oxidoreductase chain E | 2663,88 | A9HRT6 | 6 | 53159 | 56422 | 56907 | 43346 | 47123 | 44590 | 0,00 | 0,81 |
| Succinate dehydrogenase iron-sulfur subunit | 9088,36 | A9HFD5 | 11 | 146948 | 132625 | 189238 | 101854 | 140982 | 138024 | 0,12 | 0,81 |
| Peptidase, family M16 | 1172,59 | A9HKF0 | 5 | 64326 | 52675 | 34891 | 38103 | 35076 | 50510 | 0,20 | 0,81 |
| Aminotransferase | 10404,64 | A9HSE9 | 16 | 222741 | 200745 | 198129 | 158334 | 144009 | 205537 | 0,08 | 0,82 |
| Peptidyl-prolyl cis-trans isomerase | 6425,66 | A9HIQ1 | 6 | 132892 | 134994 | 126516 | 90487 | 121307 | 111824 | 0,06 | 0,82 |
| Acetyltransferase | 2747,81 | A9HJB2 | 14 | 101643 | 94002 | 68681 | 95013 | 44618 | 78276 | 0,22 | 0,82 |
| Chaperone protein HtpG | 3224,06 | A9HLJ9 | 20 | 98336 | 89580 | 113002 | 95782 | 63248 | 89270 | 0,11 | 0,83 |
| Aspartokinase | 1560,40 | A9HJ44 | 11 | 57017 | 38613 | 68246 | 44450 | 27715 | 63223 | 0,26 | 0,83 |
| Putative aminotransferase | 1301,21 | A9HD05 | 5 | 27503 | 26557 | 38359 | 28101 | 27355 | 20926 | 0,15 | 0,83 |
| 3-oxoacyl-[acyl-carrier-protein] reductase | 2263,69 | A9HRE0 | 7 | 75044 | 64892 | 104773 | 75079 | 50842 | 76640 | 0,20 | 0,83 |
| Putative multidrug resistance protein mdtA | 609,47 | A9H3E8 | 2 | 50604 | 55532 | 39962 | 48069 | 34789 | 39020 | 0,13 | 0,83 |
| Uncharacterized protein | 1578,02 | A9H3Z8 | 2 | 9894 | 10549 | 13952 | 8748 | 9007 | 10997 | 0,14 | 0,84 |
| Cysteine synthase | 3025,13 | A9HFX5 | 9 | 40226 | 38564 | 47845 | 36790 | 32160 | 36969 | 0,06 | 0,84 |
| Biopolymer transport exbB protein | 8050,85 | A9HF70 | 8 | 378848 | 437917 | 400346 | 272246 | 355287 | 391689 | 0,10 | 0,84 |
| TonB-dependent receptor | 1896,47 | A9H7L9 | 10 | 36865 | 63308 | 73865 | 53692 | 45032 | 47461 | 0,25 | 0,84 |
| Glucose-1-phosphate thymidylyltransferase | 1311,00 | A9HH12 | 7 | 37177 | 31299 | 43396 | 33413 | 31660 | 29152 | 0,11 | 0,84 |
| Aminopeptidase | 1031,12 | A9HMM0 | 6 | 45838 | 52325 | 57885 | 53053 | 37798 | 41081 | 0,12 | 0,85 |
| Threonine synthase | 1238,35 | A9HKE7 | 8 | 27083 | 26766 | 28645 | 23708 | 15305 | 30788 | 0,22 | 0,85 |
| Uridylate kinase | 3916,06 | A9HKW8 | 6 | 69598 | 62173 | 97078 | 60230 | 46030 | 87590 | 0,26 | 0,85 |
| Carbonic anhydrase | 9831,35 | A9HL77 | 10 | 142605 | 142415 | 147231 | 122515 | 131873 | 113473 | 0,02 | 0,85 |
| Lipase protein | 2665,82 | A9HBK6 | 4 | 36619 | 34351 | 34377 | 24005 | 29509 | 36223 | 0,14 | 0,85 |
| Deoxyuridine 5'-triphosphate nucleotidohydrolase | 838,35 | A9HI24 | 2 | 37714 | 30536 | 53721 | 31840 | 42539 | 29602 | 0,25 | 0,85 |
| FAD linked oxidase domain protein | 565,49 | A9H1K4 | 2 | 27108 | 12527 | 14064 | 17410 | 19668 | 8793 | 0,34 | 0,85 |
| Methylthioribose-1-phosphate isomerase | 816,50 | A9HLJ6 | 4 | 52812 | 53267 | 37020 | 33408 | 51863 | 37314 | 0,21 | 0,86 |
| Trigger factor | 27767,47 | TIG | 32 | 431991 | 434234 | 596591 | 459325 | 355861 | 438401 | 0,17 | 0,86 |
| Succinate-semialdehyde dehydrogenase [NADP+] | 1258,06 | A9HNC1 | 10 | 35479 | 31582 | 31377 | 31449 | 25846 | 27159 | 0,05 | 0,86 |
| 6,7-dimethyl-8-ribityllumazine synthase | 3493,53 | A9HDF5 | 4 | 33884 | 43834 | 25032 | 35918 | 22135 | 30229 | 0,26 | 0,86 |
| Partitioning protein | 2547,46 | A9HT61 | 3 | 42608 | 37961 | 55112 | 41001 | 36535 | 39098 | 0,17 | 0,86 |
| Bifunctional purine biosynthesis protein PurH | 3713,72 | A9HDN9 | 12 | 122411 | 97393 | 103598 | 90112 | 73728 | 114697 | 0,18 | 0,86 |
| Glycine cleavage system aminomethyltransferase T | 3855,60 | A9HM51 | 14 | 76869 | 88258 | 73043 | 60298 | 56023 | 88853 | 0,20 | 0,86 |
| Conserved protein | 6029,52 | A9HSH2 | 6 | 96608 | 101835 | 79780 | 71217 | 68077 | 100875 | 0,19 | 0,86 |
| Carboxy-terminal protease protein | 6976,38 | A9H3A3 | 16 | 121575 | 137790 | 161782 | 116419 | 82924 | 164386 | 0,26 | 0,86 |
| 2,3,4,5-tetrahydropyridine | 5734,64 | A9HKR5 | 12 | 63409 | 61658 | 94659 | 70720 | 44137 | 75238 | 0,27 | 0,87 |
| Ribosome maturation factor RimP | 7672,37 | RIMP | 8 | 57247 | 48549 | 77033 | 49171 | 54316 | 54807 | 0,22 | 0,87 |
| NADH dehydrogenase (Ubiquinone) | 2449,25 | A9HKL6 | 9 | 35347 | 36138 | 35447 | 28277 | 29515 | 34871 | 0,07 | 0,87 |
| Methionine--tRNA ligase | 1083,92 | A9HIA3 | 8 | 22566 | 32707 | 43111 | 24230 | 25944 | 35236 | 0,28 | 0,87 |
| 3-isopropylmalate dehydratase large subunit | 1926,34 | A9HS56 | 9 | 51698 | 38384 | 48965 | 41376 | 38998 | 40344 | 0,13 | 0,87 |
| Uncharacterized protein | 5674,24 | A9H3X9 | 8 | 140269 | 147078 | 146587 | 120964 | 111593 | 145058 | 0,10 | 0,87 |
| Fructose-1,6-bisphosphatase | 5516,58 | A9HCQ2 | 11 | 191127 | 155735 | 162697 | 152269 | 119924 | 172007 | 0,16 | 0,87 |
| Putative mannose-1-phosphate guanylyltransferase | 2548,52 | A9HB78 | 8 | 78954 | 91901 | 87586 | 86933 | 69892 | 68716 | 0,10 | 0,87 |
| Putative ribitol 2-dehydrogenase | 6879,68 | A9HPG2 | 10 | 139493 | 144095 | 125212 | 114561 | 147674 | 95977 | 0,19 | 0,88 |
| Cell division protein FtsZ | 7135,35 | A9H0K4 | 18 | 147346 | 150883 | 166495 | 125705 | 115996 | 165606 | 0,17 | 0,88 |
| Gluconate 2-dehydrogenase (Acceptor) | 8024,58 | A9HK15 | 14 | 274060 | 266113 | 289220 | 240619 | 237080 | 249917 | 0,01 | 0,88 |
| Elongation factor P | 5501,27 | EFP | 2 | 192885 | 103996 | 114463 | 114543 | 124749 | 121653 | 0,31 | 0,88 |
| Putative glutamyl-tRNA amidotransferase subunit A | 1333,14 | A9HJR7 | 5 | 44702 | 49606 | 58032 | 53931 | 36807 | 42983 | 0,19 | 0,88 |
| Ribonucleoside-diphosphate reductase subunit beta | 1345,01 | A9HIG8 | 5 | 21893 | 23943 | 26307 | 24310 | 15028 | 24085 | 0,23 | 0,88 |
| Flavin oxidoreductase | 1287,51 | A9H2N2 | 5 | 50481 | 53668 | 53905 | 55759 | 31757 | 51614 | 0,24 | 0,88 |
| Transcriptional regulator protein | 705,89 | A9HF00 | 2 | 41044 | 35965 | 40880 | 38077 | 41483 | 24914 | 0,24 | 0,89 |
| UTP--glucose-1-phosphate uridylyltransferase | 6071,02 | A9HJ15 | 13 | 96187 | 83199 | 86464 | 91117 | 72517 | 72121 | 0,13 | 0,89 |
| Probable cytosol aminopeptidase | 2101,75 | A9H1J4 | 10 | 70987 | 75029 | 58080 | 61896 | 51842 | 67455 | 0,16 | 0,89 |
| ATP synthase subunit beta | 23125,04 | ATPB | 28 | 358640 | 341367 | 436313 | 367765 | 304454 | 336833 | 0,15 | 0,89 |
| L-allo-threonine dehydrogenase | 2695,91 | A9H4E8 | 5 | 74467 | 73096 | 106795 | 85421 | 63112 | 77431 | 0,25 | 0,89 |
| Putaive gamma-glutamyltranspeptidase | 987,96 | A9H4A8 | 4 | 19890 | 13574 | 18667 | 13790 | 17036 | 15551 | 0,22 | 0,89 |
| Phosphoglycerate mutase | 5180,77 | A9HBZ6 | 3 | 57762 | 41856 | 46473 | 54553 | 38643 | 36918 | 0,25 | 0,89 |
| Phosphoribosylamine--glycine ligase | 3205,68 | A9H4P1 | 9 | 56232 | 59189 | 52998 | 50511 | 37518 | 61995 | 0,24 | 0,89 |
| Acetyl-coenzyme A carboxylase carboxyl transferase subunit alpha | 3577,23 | ACCA | 9 | 110153 | 99712 | 116404 | 104900 | 84016 | 104250 | 0,13 | 0,90 |
| Site-determining protein | 9094,36 | A9HLY3 | 14 | 169141 | 183269 | 153469 | 145844 | 159978 | 149580 | 0,09 | 0,90 |
| Glucokinase protein | 8096,97 | A9HIS0 | 8 | 108683 | 104707 | 125308 | 93423 | 86359 | 125641 | 0,24 | 0,90 |
| Outer membrane protein assembly factor BamD | 944,81 | A9H0L0 | 5 | 18002 | 19292 | 36434 | 14237 | 25303 | 27008 | 0,38 | 0,90 |
| Signal peptidase I | 589,38 | A9HKX7 | 2 | 20093 | 17317 | 15246 | 16959 | 15233 | 15570 | 0,18 | 0,91 |
| Putative quinoprotein glucose dehydrogenase | 470,50 | A9H181 | 5 | 24492 | 25462 | 20554 | 18970 | 16955 | 28159 | 0,31 | 0,91 |
| Phosphomannomutase alpha/beta/alpha domain I | 2112,95 | A9H070 | 12 | 67131 | 64597 | 62039 | 62302 | 47293 | 66654 | 0,21 | 0,91 |
| Efflux transporter, RND family, MFP subunit | 855,82 | A9HEF9 | 6 | 27533 | 26630 | 29595 | 26051 | 27855 | 22309 | 0,13 | 0,91 |
| Putative pyruvate dehydrogenase E1 | 2617,27 | A9HHP7 | 11 | 83345 | 79782 | 93742 | 65178 | 81816 | 88316 | 0,22 | 0,92 |
| Malate dehydrogenase ( | 1539,15 | A9HH05 | 7 | 38764 | 50378 | 30964 | 26631 | 41960 | 41452 | 0,34 | 0,92 |
| Pyruvate kinase | 11224,30 | A9HEH3 | 20 | 338979 | 320850 | 279290 | 262595 | 284107 | 314438 | 0,16 | 0,92 |
| 4-hydroxy-3-methylbut-2-enyl diphosphate | 2179,47 | A9HS93 | 7 | 42544 | 45546 | 83255 | 57010 | 43913 | 56667 | 0,38 | 0,92 |
| Aldose 1-epimerase | 1040,10 | A9HBF6 | 6 | 35420 | 50533 | 61307 | 34409 | 50225 | 51490 | 0,36 | 0,92 |
| Uncharacterized protein | 1380,16 | A9HM07 | 2 | 47607 | 41684 | 19959 | 30093 | 37453 | 33504 | 0,39 | 0,92 |
| Ribosome-recycling factor | 9105,32 | RRF | 11 | 128464 | 142068 | 189472 | 115027 | 195664 | 115336 | 0,37 | 0,93 |
| TonB-dependent receptor | 941,16 | A9HFL0 | 11 | 32357 | 43836 | 47849 | 40973 | 33243 | 40669 | 0,30 | 0,93 |
| 4-hydroxy-tetrahydrodipicolinate synthase | 4996,53 | DAPA | 13 | 74428 | 82967 | 84137 | 84711 | 68121 | 70923 | 0,19 | 0,93 |
| Chaperone protein DnaK | 25569,58 | DNAK | 42 | 562851 | 574974 | 538962 | 555559 | 486871 | 513578 | 0,09 | 0,93 |
| Cof-like hydrolase | 5334,91 | A9H332 | 10 | 111391 | 114024 | 109499 | 90634 | 100482 | 119841 | 0,23 | 0,93 |
| Putative exported protein | 3457,55 | A9H7E6 | 6 | 78793 | 80298 | 70759 | 67523 | 66362 | 79598 | 0,18 | 0,93 |
| Putative transporter protein | 1896,41 | A9H3U2 | 9 | 65793 | 59546 | 57058 | 55526 | 48580 | 65674 | 0,25 | 0,93 |
| Alkyl hydroperoxide reductase AhpD | 6980,74 | AHPD | 7 | 69738 | 65416 | 80519 | 59797 | 51369 | 89626 | 0,36 | 0,93 |
| Peptidoglycan-associated protein | 31018,29 | A9HB05 | 12 | 630651 | 676438 | 685677 | 627894 | 603531 | 627484 | 0,05 | 0,93 |
| Uncharacterized protein | 3804,50 | A9HF96 | 10 | 126809 | 133324 | 134636 | 105900 | 84843 | 177906 | 0,39 | 0,93 |
| 3-oxoacyl-[acyl-carrier-protein] reductase | 10580,89 | A9HPF0 | 11 | 241875 | 237682 | 175938 | 195955 | 197329 | 218943 | 0,29 | 0,93 |
| Phosphate import ATP-binding protein PstB | 1008,04 | A9H9W2 | 5 | 24668 | 17830 | 18925 | 15872 | 22286 | 19217 | 0,33 | 0,93 |
| UDP-glucose 6-dehydrogenase | 872,30 | A9HDC8 | 7 | 24076 | 20544 | 23810 | 23251 | 14971 | 25778 | 0,35 | 0,94 |
| Tryptophan synthase alpha chain | 1913,15 | TRPA | 4 | 29146 | 26341 | 25771 | 25323 | 26704 | 24128 | 0,13 | 0,94 |
| Type II and III secretion system protein | 1129,53 | A9HHL5 | 4 | 30833 | 24765 | 24191 | 21665 | 28191 | 24938 | 0,29 | 0,94 |
| Dipeptidyl-peptidase | 2897,88 | A9H090 | 21 | 83271 | 68639 | 99345 | 70585 | 59587 | 105471 | 0,38 | 0,94 |
| Outer membrane efflux protein | 923,15 | A9H3U0 | 7 | 25596 | 23350 | 21535 | 16813 | 21515 | 28057 | 0,36 | 0,94 |
| Bifunctional protein FolD | 1842,54 | FOLD | 6 | 30911 | 28968 | 36532 | 29530 | 30166 | 31112 | 0,25 | 0,94 |
| Conserved protein | 3893,29 | A9HRX3 | 10 | 52172 | 63930 | 65699 | 44844 | 53576 | 72824 | 0,37 | 0,94 |
| Indole-3-glycerol phosphate synthase | 2300,52 | A9HJA0 | 6 | 36285 | 32684 | 37783 | 35710 | 32537 | 32322 | 0,17 | 0,94 |
| Gluconate 5-dehydrogenase | 10920,20 | A9H995 | 12 | 151593 | 169085 | 200631 | 158318 | 163691 | 169284 | 0,28 | 0,94 |
| 10 kDa chaperonin | 43950,11 | A9HK46 | 9 | 624357 | 602612 | 601471 | 624022 | 505530 | 594300 | 0,22 | 0,94 |
| Putative polysaccharide export protein | 1050,99 | A9HMV6 | 8 | 65517 | 64457 | 52926 | 58174 | 57001 | 57389 | 0,24 | 0,94 |
| Transketolase | 19952,19 | A9H317 | 36 | 434869 | 478454 | 504160 | 477153 | 353669 | 510049 | 0,33 | 0,95 |
| Phosphoribosylformylglycinamidine synthase | 2510,93 | A9HJG3 | 19 | 45067 | 65557 | 55584 | 53499 | 30695 | 73113 | 0,42 | 0,95 |
| TonB-dependent Receptor protein | 7995,05 | A9HFV5 | 31 | 323266 | 352993 | 312331 | 308021 | 271967 | 355737 | 0,28 | 0,95 |
| Enoyl-[acyl-carrier-protein] reductase [NADH] | 2534,02 | A9H0U5 | 7 | 89036 | 100968 | 104977 | 89125 | 74688 | 115408 | 0,36 | 0,95 |
| 3-isopropylmalate dehydratase small subunit | 2177,73 | LEUD | 6 | 13309 | 21354 | 64136 | 20554 | 14462 | 58856 | 0,47 | 0,95 |
| ATP synthase epsilon chain | 1762,54 | ATPE | 3 | 37977 | 36082 | 47187 | 26384 | 61080 | 27817 | 0,44 | 0,95 |
| Protein-L-isoaspartate | 5076,88 | A9HI07 | 7 | 118233 | 85697 | 93665 | 85165 | 96226 | 101594 | 0,34 | 0,95 |
| DNA-directed RNA polymerase subunit alpha | 25067,62 | RPOA | 20 | 395405 | 376923 | 422140 | 380902 | 377097 | 380990 | 0,15 | 0,95 |
| Putative dihydro-orotase protein | 1002,10 | A9GZR4 | 6 | 25840 | 24601 | 21319 | 30396 | 14870 | 23235 | 0,42 | 0,95 |
| Conjugal transfer | 9616,33 | A9HT68 | 8 | 72606 | 73413 | 94985 | 78099 | 69097 | 83034 | 0,35 | 0,96 |
| DNA-binding protein HU | 54597,21 | A9HRU7 | 5 | 454996 | 433850 | 402367 | 470828 | 268993 | 495478 | 0,41 | 0,96 |
| Bifunctional enzyme IspD/IspF | 2260,44 | A9HLU2 | 10 | 72430 | 67651 | 62149 | 64231 | 51149 | 79328 | 0,40 | 0,96 |
| RNA-binding protein Hfq | 12983,29 | HFQ | 7 | 104009 | 95897 | 113405 | 98898 | 93922 | 109322 | 0,31 | 0,96 |
| Cof-like hydrolase | 1042,54 | A9H329 | 5 | 32233 | 21214 | 32462 | 29188 | 27282 | 26470 | 0,41 | 0,97 |
| Putative aerobic cobaltochelatase cobS subunit | 2951,74 | A9HJ01 | 10 | 81967 | 94006 | 115248 | 94559 | 70664 | 116485 | 0,43 | 0,97 |
| S-methyl-5'-thioadenosine phosphorylase | 5078,06 | A9HK57 | 10 | 56425 | 82233 | 71795 | 63134 | 57769 | 83035 | 0,42 | 0,97 |
| Protein RecA | 4068,10 | A9HM16 | 13 | 93693 | 97071 | 99653 | 88214 | 101343 | 92429 | 0,28 | 0,97 |
| Protein TonB | 4261,60 | A9HF68 | 6 | 113053 | 79682 | 131070 | 105284 | 98396 | 111192 | 0,43 | 0,97 |
| PEBP family protein | 2148,95 | A9HBI3 | 3 | 39307 | 37575 | 73632 | 51243 | 60855 | 34294 | 0,46 | 0,97 |
| Heat shock protein Hsp20 | 6391,58 | A9HHR8 | 7 | 76473 | 60392 | 127852 | 84767 | 62906 | 110587 | 0,47 | 0,98 |
| Branched-chain-amino-acid aminotransferase | 7157,15 | A9HNB7 | 16 | 131092 | 141742 | 159337 | 149534 | 104609 | 167777 | 0,44 | 0,98 |
| Uroporphyrinogen decarboxylase | 1988,70 | DCUP | 8 | 38901 | 40550 | 40706 | 35403 | 37575 | 44568 | 0,39 | 0,98 |
| Thiol specific antioxidant/ Mal allergen | 44334,10 | A9H8D6 | 17 | 471631 | 474215 | 540590 | 441389 | 459936 | 553341 | 0,41 | 0,98 |
| Glutamate synthase [NADPH] small chain | 3807,90 | A9HKL4 | 13 | 59405 | 50194 | 55143 | 55025 | 40518 | 65748 | 0,45 | 0,98 |
| ATP synthase subunit alpha | 22091,75 | ATPA | 30 | 354971 | 372014 | 344930 | 348809 | 322778 | 378084 | 0,35 | 0,98 |
| Malonyl CoA-acyl carrier protein transacylase | 4618,99 | A9HRE1 | 9 | 60495 | 79476 | 72227 | 66591 | 56617 | 84732 | 0,45 | 0,98 |
| Glyceraldehyde-3-phosphate dehydrogenase | 29860,08 | A9HM29 | 20 | 646421 | 649256 | 501337 | 581573 | 578351 | 601208 | 0,42 | 0,98 |
| Lipoprotein | 1480,68 | A9HPJ3 | 4 | 16225 | 14325 | 16026 | 11898 | 13912 | 19876 | 0,46 | 0,98 |
| Acetyl-CoA hydrolase | 6802,01 | A9HIK2 | 22 | 168304 | 182197 | 298863 | 229350 | 154667 | 253850 | 0,47 | 0,98 |
| Nucleoside diphosphate kinase | 15674,95 | NDK | 5 | 373753 | 357952 | 324282 | 311107 | 383692 | 345164 | 0,42 | 0,98 |
| Probable malate:quinone oxidoreductase | 1752,41 | A9HKZ6 | 8 | 31970 | 33480 | 49139 | 38130 | 32405 | 42343 | 0,47 | 0,99 |
| 1-deoxy-D-xylulose-5-phosphate synthase | 2049,58 | A9HIR0 | 15 | 57975 | 59847 | 71943 | 64913 | 50585 | 71762 | 0,46 | 0,99 |
| Putative toluene tolerance | 6657,56 | A9HBF1 | 4 | 130975 | 84648 | 92719 | 86068 | 126603 | 91665 | 0,47 | 0,99 |
| Putative outer membrane protein | 5822,32 | A9H4M9 | 7 | 102313 | 103600 | 103359 | 89963 | 100946 | 114645 | 0,44 | 0,99 |
| Aminopeptidase | 7730,23 | A9HFU5 | 31 | 167054 | 173888 | 210073 | 194019 | 145944 | 204568 | 0,46 | 0,99 |
| Signal recognition particle receptor FtsY | 1118,03 | A9HM31 | 6 | 18658 | 20001 | 21275 | 14207 | 16783 | 28245 | 0,48 | 0,99 |
| Ubiquinone biosynthesis | 1607,68 | A9HJ43 | 4 | 19853 | 23220 | 28442 | 22553 | 25222 | 22943 | 0,46 | 0,99 |
| 30S ribosomal protein S9 | 3835,83 | A9H812 | 6 | 57663 | 62794 | 77041 | 82964 | 30107 | 82485 | 0,49 | 0,99 |
| ATP-dependent Clp protease proteolytic subunit | 11451,86 | A9HCR1 | 11 | 236854 | 240711 | 274002 | 238218 | 260447 | 250019 | 0,47 | 1,00 |
| D-mannonate oxidoreductase | 782,06 | A9H179 | 5 | 36950 | 34750 | 26651 | 32278 | 38294 | 27406 | 0,49 | 1,00 |
| Aspartyl/glutamyl-tRNA amidotransferase subunit B | 1825,80 | A9HRI7 | 15 | 67633 | 65090 | 79731 | 68940 | 60600 | 82222 | 0,49 | 1,00 |
| Dihydrolipoyllysine-residue succinyltransferase | 12004,75 | A9HFG9 | 14 | 306604 | 321319 | 318687 | 290031 | 344933 | 308908 | 0,48 | 1,00 |
| OmpW family protein | 20385,57 | A9HED6 | 7 | 370994 | 402630 | 393640 | 389989 | 402393 | 374043 | 0,49 | 1,00 |
| Inositol-1-monophosphatase | 1154,17 | A9HRD4 | 2 | 31936 | 33372 | 27589 | 26876 | 36110 | 29854 | 0,50 | 1,00 |
| Uncharacterized protein | 1096,22 | A9H0Y9 | 5 | 42886 | 35278 | 33075 | 37420 | 37348 | 36561 | 0,50 | 1,00 |
| Cold shock-like protein cspE | 21949,05 | A9HIW8 | 12 | 610078 | 555183 | 567723 | 543721 | 566922 | 627265 | 0,48 | 1,00 |
| Protein-export protein SecB | 10530,74 | SECB | 8 | 159171 | 154443 | 212352 | 170341 | 166536 | 190763 | 0,49 | 1,00 |
| Serine protease | 5320,28 | A9HEK6 | 19 | 195122 | 209255 | 198521 | 203063 | 185485 | 216371 | 0,48 | 1,00 |
| Chaperone SurA | 1164,54 | A9H1L4 | 5 | 43664 | 52378 | 40489 | 43511 | 34865 | 58689 | 0,49 | 1,00 |
| Isocitrate dehydrogenase (NAD(+)) | 12373,39 | A9HJQ1 | 14 | 383162 | 384956 | 360979 | 377065 | 400303 | 356192 | 0,46 | 1,00 |
| 50S ribosomal protein L9 | 26520,55 | RL9 | 13 | 411916 | 421511 | 361250 | 386593 | 414719 | 398327 | 0,47 | 1,00 |
| Arginine--tRNA ligase | 1226,14 | A9HLH8 | 8 | 21768 | 24263 | 24784 | 31041 | 15224 | 24958 | 0,49 | 1,01 |
| Biotin synthase | 1086,73 | BIOB | 4 | 39708 | 31128 | 30717 | 30878 | 26272 | 45172 | 0,49 | 1,01 |
| Methylenetetrahydrofolate reductase | 1795,17 | A9HNY2 | 9 | 25311 | 23923 | 31083 | 23173 | 30668 | 27419 | 0,46 | 1,01 |
| Electron transfer flavoprotein alpha subunit | 5739,78 | A9HEE9 | 10 | 151818 | 146193 | 137663 | 158686 | 130169 | 151938 | 0,43 | 1,01 |
| D-2-hydroxyacid dehydrogensase | 20482,92 | A9HDT4 | 15 | 342981 | 317556 | 335817 | 379369 | 304365 | 324747 | 0,44 | 1,01 |
| 60 kDa chaperonin 1 | 38262,66 | CH601 | 43 | 671974 | 672055 | 825044 | 741156 | 701396 | 753955 | 0,44 | 1,01 |
| Acetoin reductase | 1488,82 | A9HPB2 | 4 | 57744 | 47824 | 41811 | 38530 | 72132 | 38589 | 0,48 | 1,01 |
| Beta sliding clamp | 3524,14 | A9HI34 | 17 | 88389 | 80430 | 107509 | 93735 | 99298 | 86841 | 0,45 | 1,01 |
| Pyruvate dehydrogenase E1 component subunit beta | 4216,06 | A9HJA9 | 14 | 107962 | 106104 | 93407 | 116741 | 70920 | 124214 | 0,47 | 1,01 |
| Phosphoribosylformylglycinamidine synthase | 1260,84 | A9HJG0 | 5 | 66201 | 68419 | 53285 | 54940 | 53305 | 82761 | 0,46 | 1,02 |
| Glycosyl transferase group 1 | 2403,67 | A9HLZ7 | 11 | 58110 | 73901 | 50221 | 54955 | 42344 | 88124 | 0,47 | 1,02 |
| Uncharacterized protein | 2150,84 | A9H8I6 | 8 | 48864 | 45065 | 47798 | 39731 | 54617 | 50232 | 0,43 | 1,02 |
| 50S ribosomal protein L19 | 5760,80 | RL19 | 3 | 40140 | 34648 | 53933 | 49827 | 33708 | 48005 | 0,45 | 1,02 |
| 50S ribosomal protein L10 | 34011,68 | RL10 | 11 | 388594 | 382673 | 340055 | 359721 | 389907 | 386297 | 0,34 | 1,02 |
| Phosphoribosylformylglycinamidine cyclo-ligase | 4713,23 | A9HJV0 | 9 | 91373 | 82306 | 79322 | 79696 | 79862 | 99075 | 0,41 | 1,02 |
| Putative nitrogen fixation protein | 5874,71 | A9HFA7 | 7 | 106287 | 102545 | 98610 | 104945 | 92255 | 117500 | 0,39 | 1,02 |
| Aspartate-semialdehyde dehydrogenase | 6983,36 | A9HFE5 | 17 | 105007 | 110001 | 107761 | 81345 | 84012 | 165445 | 0,47 | 1,02 |
| Mannitol 2-dehydrogenase | 2354,43 | A9HBL5 | 15 | 51111 | 59269 | 52137 | 52973 | 36135 | 77689 | 0,46 | 1,03 |
| Citrate synthase | 6469,10 | A9HII7 | 18 | 114680 | 133733 | 172627 | 156480 | 112883 | 162874 | 0,44 | 1,03 |
| ATP phosphoribosyltransferase regulatory subunit | 1512,60 | A9HLQ2 | 4 | 16899 | 18672 | 23839 | 25968 | 16233 | 18821 | 0,44 | 1,03 |
| 2-amino-3-ketobutyrate coenzyme A ligase | 537,17 | A9HRC0 | 3 | 15315 | 14092 | 19868 | 16941 | 14281 | 19445 | 0,43 | 1,03 |
| BolA family protein | 7704,89 | A9HJG6 | 3 | 59660 | 47540 | 55867 | 41853 | 59815 | 66013 | 0,43 | 1,03 |
| Acetolactate synthase | 2043,40 | A9GZI8 | 11 | 52168 | 48455 | 60959 | 46934 | 53787 | 65478 | 0,41 | 1,03 |
| Ferredoxin | 7008,81 | A9HJZ0 | 3 | 159557 | 139559 | 147870 | 176896 | 176438 | 106917 | 0,43 | 1,03 |
| Nicotinate-nucleotide pyrophosphorylase | 4037,65 | A9H8C5 | 9 | 66634 | 75537 | 100861 | 79662 | 76881 | 93763 | 0,42 | 1,03 |
| Putative molybdenum transport protein modE | 38203,03 | A9HJC5 | 5 | 327655 | 334916 | 331201 | 355925 | 317582 | 350172 | 0,25 | 1,03 |
| UDP-3--acylglucosamine N-acyltransferase | 1868,74 | A9HKU5 | 9 | 125610 | 129342 | 48509 | 134270 | 25325 | 153179 | 0,48 | 1,03 |
| Tryptophan synthase beta chain | 1461,45 | A9HE87 | 7 | 37136 | 36690 | 57079 | 54088 | 39182 | 41660 | 0,44 | 1,03 |
| Thioredoxin protein | 3990,63 | A9H2A4 | 8 | 67592 | 63852 | 75379 | 68780 | 79160 | 65371 | 0,35 | 1,03 |
| FeS assembly protein SufC | 1896,28 | A9HRY6 | 8 | 27364 | 29161 | 31540 | 36142 | 29923 | 24961 | 0,40 | 1,03 |
| Inositol-3-phosphate synthase | 23448,24 | A9H8S7 | 17 | 290293 | 352989 | 472312 | 369390 | 294719 | 489985 | 0,44 | 1,03 |
| Outer membrane protein | 19558,81 | OMPC | 17 | 750299 | 745990 | 641876 | 713776 | 624955 | 877876 | 0,39 | 1,04 |
| L-idonate 5-dehydrogenase | 2159,72 | A9HDU6 | 11 | 78881 | 87047 | 61723 | 70023 | 70479 | 95676 | 0,41 | 1,04 |
| Elongation factor Ts | 15029,59 | A9HRQ5 | 16 | 270066 | 278193 | 297172 | 283669 | 230714 | 362718 | 0,41 | 1,04 |
| 6-phosphogluconolactonase | 8259,89 | A9H335 | 9 | 151264 | 158362 | 157793 | 147868 | 142857 | 194327 | 0,38 | 1,04 |
| Enolase | 24885,39 | ENO | 22 | 432612 | 413535 | 372060 | 413558 | 393747 | 458547 | 0,29 | 1,04 |
| Putative glycyl aminopeptidase | 3102,00 | A9HN12 | 19 | 90640 | 108325 | 131057 | 134057 | 87157 | 122307 | 0,41 | 1,04 |
| 2-ketogluconate reductase | 1233,72 | A9H3Y4 | 6 | 31167 | 29388 | 39606 | 34021 | 35473 | 35001 | 0,35 | 1,04 |
| Alcohol dehydrogenase [acceptor] | 7714,38 | A9HK12 | 26 | 257303 | 267135 | 255961 | 257096 | 248036 | 309728 | 0,31 | 1,04 |
| Carbamoyl-phosphate synthase small chain | 2830,57 | A9H1P4 | 10 | 32694 | 38506 | 50205 | 32406 | 26610 | 67765 | 0,45 | 1,04 |
| Surface antigen protein | 5233,49 | A9HAP4 | 5 | 167378 | 172943 | 127759 | 147932 | 167888 | 173087 | 0,35 | 1,04 |
| Fructose-bisphosphate aldolase class 1 | 11035,31 | A9H6A5 | 14 | 153441 | 150246 | 174793 | 137982 | 168022 | 195049 | 0,35 | 1,05 |
| Glutamine--fructose-6-phosphate aminotransferase | 788,98 | A9HI49 | 7 | 18136 | 21933 | 21896 | 22301 | 11751 | 30950 | 0,44 | 1,05 |
| 50S ribosomal protein L11 | 22286,92 | RL11 | 13 | 395200 | 409272 | 439990 | 417170 | 394835 | 498204 | 0,29 | 1,05 |
| 3-oxoacyl-[acyl-carrier-protein] reductase | 4922,67 | A9HIY5 | 8 | 61331 | 74351 | 84496 | 100529 | 46887 | 84676 | 0,42 | 1,05 |
| Phosphoglycerate kinase | 12815,05 | A9HM30 | 19 | 306230 | 270244 | 311164 | 304508 | 301784 | 332481 | 0,18 | 1,06 |
| 3-isopropylmalate dehydrogenase | 1345,31 | A9HS52 | 6 | 26703 | 45154 | 32320 | 46595 | 29630 | 34124 | 0,40 | 1,06 |
| Superoxide dismutase | 12063,78 | A9HL14 | 11 | 339882 | 336240 | 332234 | 354068 | 323852 | 398935 | 0,20 | 1,07 |
| Inorganic pyrophosphatase | 8866,39 | A9H4G5 | 6 | 207963 | 235440 | 232226 | 232953 | 184375 | 309777 | 0,34 | 1,08 |
| Beta-lactamase-like protein | 1762,23 | A9HC31 | 5 | 11550 | 13488 | 13543 | 12590 | 11652 | 17398 | 0,32 | 1,08 |
| Bacterioferritin | 8036,52 | A9H7G6 | 10 | 78278 | 81691 | 95806 | 72843 | 92802 | 110833 | 0,31 | 1,08 |
| NADH-quinone oxidoreductase subunit B 2 | 3849,32 | NUOB2 | 4 | 33535 | 35793 | 25770 | 22457 | 37153 | 43351 | 0,37 | 1,08 |
| S-adenosylmethionine synthase | 12607,16 | METK | 22 | 232507 | 280358 | 294901 | 284093 | 234572 | 356092 | 0,31 | 1,08 |
| Flavin-dependent thymidylate synthase | 1534,15 | A9HBG1 | 6 | 24159 | 25379 | 29366 | 33458 | 12890 | 39461 | 0,40 | 1,09 |
| Protein GrpE | 3677,90 | A9HEA5 | 9 | 125743 | 119567 | 122517 | 98675 | 118486 | 183768 | 0,35 | 1,09 |
| Putative pyruvate dehydrogenase E2 component | 1381,95 | A9HHP4 | 7 | 56320 | 64283 | 40990 | 70189 | 60267 | 45759 | 0,32 | 1,09 |
| ATP synthase subunit delta | 5526,72 | ATPD | 8 | 51161 | 46837 | 52826 | 51740 | 57953 | 55473 | 0,07 | 1,10 |
| Succinate-semialdehyde dehydrogenase | 12088,71 | A9H549 | 27 | 288394 | 279662 | 257723 | 347820 | 294640 | 263383 | 0,20 | 1,10 |
| Acetylornithine aminotransferase | 6138,89 | A9HFT8 | 16 | 91361 | 95759 | 89119 | 85104 | 86217 | 131908 | 0,31 | 1,10 |
| Serine hydroxymethyltransferase | 5294,72 | A9HRP5 | 15 | 113963 | 100739 | 123507 | 123179 | 124610 | 125125 | 0,11 | 1,10 |
| L-threonine aldolase | 725,41 | A9HMZ5 | 2 | 13508 | 15199 | 23570 | 20950 | 16971 | 19781 | 0,32 | 1,10 |
| ATP synthase gamma chain | 2738,09 | ATPG | 9 | 40289 | 42868 | 53603 | 44008 | 60458 | 46746 | 0,25 | 1,11 |
| Thioredoxin | 30565,09 | A9HA92 | 4 | 240178 | 244681 | 301280 | 308521 | 288944 | 272661 | 0,15 | 1,11 |
| Putative penicillin-binding protein | 891,47 | A9H435 | 3 | 14356 | 10567 | 12687 | 13376 | 15230 | 13063 | 0,18 | 1,11 |
| Peptidyl-prolyl cis-trans isomerase | 22006,13 | A9HM70 | 6 | 306780 | 355297 | 316425 | 348641 | 337782 | 404392 | 0,11 | 1,11 |
| Glucans biosynthesis protein G | 2836,84 | A9HBM4 | 13 | 44760 | 42897 | 65255 | 59114 | 39333 | 72212 | 0,32 | 1,12 |
| 50S ribosomal protein L15 | 11325,63 | RL15 | 4 | 133343 | 141819 | 147571 | 185293 | 148208 | 139338 | 0,18 | 1,12 |
| 50S ribosomal protein L25 | 16126,46 | RL25 | 13 | 359967 | 350061 | 346563 | 401365 | 416224 | 365072 | 0,05 | 1,12 |
| Heat shock protein Hsp20 | 3241,62 | A9HCX4 | 5 | 31300 | 34772 | 48013 | 35651 | 48047 | 44004 | 0,26 | 1,12 |
| D-3-phosphoglycerate dehydrogenase | 3200,11 | A9HIU7 | 14 | 48139 | 72385 | 60674 | 59342 | 42557 | 100972 | 0,36 | 1,12 |
| RNA-metabolising metallo-beta-lactamase protein | 4754,68 | A9HRR8 | 16 | 115001 | 120573 | 152241 | 129844 | 122623 | 181765 | 0,26 | 1,12 |
| Conserved protein | 625,20 | A9H282 | 4 | 22044 | 18841 | 25111 | 22588 | 25231 | 26192 | 0,14 | 1,12 |
| Pyridine nucleotide-disulphide oxidoreductase | 8397,64 | A9HDE6 | 15 | 102180 | 100714 | 93399 | 104347 | 107130 | 123452 | 0,08 | 1,13 |
| Putative chemotaxis protein cheY | 8308,48 | A9HHE7 | 6 | 60407 | 58312 | 56885 | 69262 | 55001 | 74806 | 0,16 | 1,13 |
| Conserved protein | 5601,80 | A9HM79 | 2 | 98549 | 102191 | 140118 | 115210 | 130441 | 140784 | 0,19 | 1,13 |
| 30S ribosomal protein S6 | 6463,84 | RS6 | 4 | 58201 | 62040 | 77564 | 86918 | 55074 | 82467 | 0,25 | 1,13 |
| Nitrogen regulatory protein P-II | 4289,21 | A9HMD4 | 5 | 58554 | 54698 | 80733 | 84257 | 82783 | 54753 | 0,25 | 1,14 |
| Aldehyde Dehydrogenase | 3398,42 | A9H4V7 | 16 | 71292 | 70604 | 103866 | 94766 | 92615 | 94732 | 0,19 | 1,15 |
| Thiamine biosynthesis oxidoreductase thi | 3138,46 | A9HI52 | 7 | 33719 | 56394 | 60008 | 59792 | 34030 | 78608 | 0,33 | 1,15 |
| Outer membrane protein | 19141,05 | A9HKU8 | 12 | 199965 | 211034 | 249272 | 254697 | 218318 | 286148 | 0,13 | 1,15 |
| Peptidase | 2557,20 | A9HEL6 | 15 | 59601 | 59283 | 52463 | 67369 | 44364 | 86538 | 0,27 | 1,16 |
| Thiazole synthase | 1412,08 | THIG | 6 | 14076 | 15133 | 23886 | 15249 | 14843 | 31355 | 0,34 | 1,16 |
| Oxidoreductase | 1356,02 | A9HAF3 | 6 | 18343 | 16843 | 22455 | 24395 | 20461 | 22040 | 0,11 | 1,16 |
| Acetylglutamate kinase | 2148,18 | ARGB | 8 | 27506 | 40292 | 46016 | 36488 | 37996 | 57774 | 0,26 | 1,16 |
| Putative haloacid dehalogenase-like hydrolase | 1251,16 | A9HBE7 | 4 | 23467 | 25949 | 17291 | 19954 | 31175 | 26604 | 0,21 | 1,17 |
| Biotin carboxyl carrier protein of acetyl-CoA | 6297,88 | A9HEX3 | 5 | 120126 | 119386 | 47442 | 94165 | 146913 | 96040 | 0,30 | 1,17 |
| Ferredoxin--NADP reductase | 3163,08 | A9HLF6 | 10 | 69544 | 72610 | 92025 | 92819 | 84799 | 97836 | 0,09 | 1,18 |
| Transcription elongation factor GreA | 7080,98 | A9H1Q0 | 7 | 91235 | 89519 | 128171 | 141341 | 112901 | 110033 | 0,16 | 1,18 |
| Polyribonucleotide nucleotidyltransferase | 13286,82 | PNP | 34 | 216122 | 217657 | 196974 | 266854 | 184034 | 295123 | 0,18 | 1,18 |
| Pyrrolo-quinoline quinone | 1676,08 | A9H134 | 6 | 64895 | 60506 | 34177 | 56167 | 63609 | 69204 | 0,21 | 1,18 |
| Adenosylhomocysteinase | 6815,86 | A9HFJ7 | 18 | 142522 | 150524 | 118531 | 173619 | 148185 | 167797 | 0,05 | 1,19 |
| Lysine--tRNA ligase | 575,15 | A9HK29 | 5 | 30765 | 28264 | 26590 | 37582 | 35279 | 29264 | 0,07 | 1,19 |
| 50S ribosomal protein L7/L12 | 38265,37 | RL7 | 5 | 256415 | 393855 | 427085 | 423275 | 245294 | 623030 | 0,30 | 1,20 |
| ROS/MUCR transcriptional regulator protein | 3301,82 | A9HFC1 | 4 | 67762 | 65755 | 67057 | 63363 | 117527 | 60053 | 0,27 | 1,20 |
| Adenylosuccinate synthetase | 4591,50 | PURA | 18 | 72026 | 74481 | 80982 | 98015 | 84760 | 91428 | 0,02 | 1,21 |
| Uncharacterized protein | 4482,90 | A9H8H8 | 4 | 46996 | 51320 | 59625 | 51628 | 80308 | 58587 | 0,17 | 1,21 |
| Tyrosine--tRNA ligase | 1039,00 | A9HMK2 | 6 | 22518 | 19208 | 20381 | 24268 | 27876 | 22779 | 0,04 | 1,21 |
| DSBA oxidoreductase | 3159,34 | A9HIK8 | 7 | 50209 | 44263 | 28422 | 64362 | 34423 | 50643 | 0,23 | 1,22 |
| NADH-quinone oxidoreductase | 3560,35 | A9HRT3 | 17 | 86238 | 107577 | 66053 | 102538 | 90374 | 123323 | 0,15 | 1,22 |
| UDP-N-acetylmuramate--L-alanine ligase | 469,23 | MURC | 2 | 27329 | 35136 | 28261 | 43260 | 32311 | 35291 | 0,09 | 1,22 |
| Putative transcriptional regulator protein | 20576,23 | A9H0C0 | 9 | 122440 | 122214 | 176908 | 147911 | 192290 | 175285 | 0,12 | 1,22 |
| Uncharacterized protein | 5482,68 | A9HAE9 | 5 | 47920 | 57072 | 56326 | 63177 | 64112 | 72450 | 0,02 | 1,24 |
| NADH-quinone oxidoreductase subunit C | 1598,77 | A9HRU1 | 3 | 28533 | 32745 | 35646 | 49138 | 31555 | 40367 | 0,12 | 1,25 |
| 50S ribosomal protein L1 | 19995,57 | RL1 | 16 | 298299 | 290743 | 281757 | 350997 | 395409 | 347912 | 0,02 | 1,26 |
| Transcription termination/antitermination protein NusA | 3257,57 | A9HF12 | 13 | 84859 | 79194 | 79509 | 115118 | 90053 | 103982 | 0,04 | 1,27 |
| Outer membrane protein assembly factor BamA | 1865,93 | A9HKV0 | 14 | 50887 | 39407 | 41837 | 41464 | 57631 | 68956 | 0,14 | 1,27 |
| Adenylate kinase | 7274,62 | KAD | 11 | 69215 | 68726 | 85017 | 98821 | 94235 | 94277 | 0,02 | 1,29 |
| ABC transporter related | 4001,29 | A9HPE7 | 9 | 36944 | 43875 | 73311 | 63198 | 51996 | 83471 | 0,18 | 1,29 |
| Single-stranded DNA-binding protein | 2665,89 | A9HM62 | 5 | 38712 | 85068 | 68490 | 64074 | 103813 | 80712 | 0,18 | 1,29 |
| Inositol-1-monophosphatase | 2674,33 | A9H6E0 | 6 | 49713 | 44242 | 56444 | 61561 | 57332 | 76013 | 0,05 | 1,30 |
| Tryptophan--tRNA ligase | 1190,14 | A9HIP8 | 6 | 12730 | 15270 | 9532 | 14853 | 19299 | 14547 | 0,09 | 1,30 |
| Homoserine dehydrogenase | 1184,46 | A9HCQ4 | 8 | 30061 | 34649 | 40743 | 50587 | 41626 | 45203 | 0,03 | 1,30 |
| Putative membrane protein | 2136,61 | A9HAA2 | 5 | 55347 | 44907 | 42405 | 42575 | 62602 | 80796 | 0,16 | 1,30 |
| Putative 3-oxoacyl-[acyl-carrier-protein] reductase | 2775,37 | A9H2M9 | 7 | 57192 | 43555 | 30833 | 53887 | 27955 | 89870 | 0,27 | 1,30 |
| Putative sulfotransferase | 937,88 | A9HQQ2 | 5 | 22951 | 23254 | 15248 | 27135 | 25092 | 28282 | 0,06 | 1,31 |
| Elongation factor Tu | 24595,95 | EFTU | 23 | 622181 | 640780 | 579046 | 805589 | 758519 | 858390 | 0,00 | 1,32 |
| 2-isopropylmalate synthase | 4022,12 | A9HMA2 | 24 | 72789 | 65537 | 54825 | 98718 | 76565 | 78767 | 0,04 | 1,32 |
| Putative rod shape-determining protein mreB | 2297,89 | A9HM98 | 9 | 51641 | 53615 | 44340 | 86271 | 30547 | 80597 | 0,23 | 1,32 |
| 30S ribosomal protein S19 | 11013,64 | RS19 | 4 | 91210 | 95760 | 131423 | 159272 | 118078 | 145867 | 0,06 | 1,33 |
| 50S ribosomal protein L4 | 8650,71 | RL4 | 9 | 129007 | 125541 | 126849 | 164352 | 187755 | 162100 | 0,02 | 1,35 |
| Putative cytochrome c551 peroxidase | 1228,86 | A9HK81 | 5 | 13363 | 24406 | 23452 | 25256 | 23910 | 33420 | 0,10 | 1,35 |
| Gluconokinase | 7172,32 | A9HSC5 | 9 | 55182 | 50922 | 76205 | 79153 | 71228 | 97568 | 0,06 | 1,36 |
| Cold-shock DEAD box protein A homolog | 2091,10 | A9HRW6 | 16 | 37563 | 32758 | 33104 | 44771 | 53917 | 43712 | 0,02 | 1,38 |
| Transcriptional regulator LysR | 4735,42 | A9H0A8 | 11 | 93743 | 69468 | 102528 | 123995 | 112777 | 133586 | 0,03 | 1,39 |
| Secretion protein, HlyD-family | 3710,43 | A9HA48 | 10 | 42404 | 33778 | 40091 | 56505 | 50990 | 54798 | 0,01 | 1,40 |
| Putative TonB-dependent receptor | 1024,28 | A9H9T7 | 5 | 32712 | 44863 | 12600 | 44400 | 31951 | 49558 | 0,17 | 1,40 |
| Quinolinate synthase A | 7769,63 | A9H8C0 | 15 | 119641 | 113851 | 125351 | 160553 | 137379 | 205566 | 0,07 | 1,40 |
| OmpA/MotB domain protein | 6138,00 | A9GZP4 | 9 | 176491 | 451732 | 175038 | 227989 | 388289 | 525308 | 0,21 | 1,42 |
| Putative L-aspartate oxidase | 465,03 | A9H8C2 | 2 | 10611 | 12338 | 13269 | 14273 | 15688 | 21555 | 0,07 | 1,42 |
| Glycosyl transferase | 7125,60 | A9HH55 | 7 | 84131 | 93069 | 97724 | 101800 | 89464 | 206967 | 0,19 | 1,45 |
| 50S ribosomal protein L18 | 1127,63 | RL18 | 2 | 16109 | 16452 | 35961 | 25353 | 40327 | 34553 | 0,13 | 1,46 |
| Trehalose 6-phosphate phosphatase | 2345,21 | A9HBU3 | 8 | 33578 | 31589 | 24895 | 47067 | 32851 | 52562 | 0,06 | 1,47 |
| Putative D-3-phosphoglycerate dehydrogenase | 543,85 | A9HFV9 | 2 | 23275 | 17184 | 20982 | 36867 | 42404 | 12011 | 0,20 | 1,49 |
| Bacteriocin protein | 2718,53 | A9H5P1 | 8 | 71020 | 71164 | 23266 | 78636 | 58610 | 108683 | 0,14 | 1,49 |
| Aspartate--tRNA(Asp/Asn) ligase | 3349,50 | A9HLJ4 | 18 | 49859 | 32339 | 79063 | 87402 | 58349 | 98270 | 0,10 | 1,51 |
| Ferrochelatase | 570,22 | A9HEQ4 | 2 | 7901 | 8062 | 14409 | 8946 | 17137 | 19891 | 0,13 | 1,51 |
| 3-phosphoshikimate 1-carboxyvinyltransferase | 2287,07 | A9H466 | 14 | 88705 | 83047 | 43921 | 55865 | 47210 | 274532 | 0,27 | 1,75 |
| Uncharacterized protein | 10008,33 | A9HH94 | 5 | 49815 | 50132 | 21376 | 60305 | 38260 | 114421 | 0,16 | 1,76 |
| Transcription termination NusG | 1170,49 | A9H988 | 4 | 19088 | 18110 | 23298 | 31676 | 64440 | 27279 | 0,11 | 2,04 |
| Zinc-binding alcohol dehydrogenase | 1686,22 | A9H246 | 9 | 29802 | 27189 | 33209 | 102302 | 24675 | 120250 | 0,11 | 2,74 |
| Catalase | 5533,55 | A9GZZ4 | 13 | 45783 | 45791 | 43201 | 117540 | 198064 | 60920 | 0,09 | 2,79 |
| Phospho-2-dehydro-3-deoxyheptonate aldolase | 710,82 | A9HLE8 | 8 | 9901 | 7541 | 8650 | 58054 | 16176 | 47555 | 0,06 | 4,67 |
| Transaldolase | 3087,37 | A9H320 | 19 | 5674 | 5999 | 3724 | 79376 | 117764 | 17391 | 0,08 | 13,93 |
| GTP-binding protein TypA/BipA | 1447,02 | A9H9C1 | 10 | 3779 | 4256 | 3457 | 62602 | 82986 | 22175 | 0,05 | 14,60 |
| Aminotransferase | 1087,26 | A9HCQ6 | 7 | 15659 | 13947 | -1 | 38277 | 59074 | 22590 | 0,04 | 4,05 |
| UPF0303 protein GDI1201 | 645,33 | A9HDU3 | 3 | -1 | -1 | 8847 | 16303 | 18456 | 6203 | 0,05 | 4,63 |
| Hopanoid-associated sugar epimerase | 908,81 | A9HGZ6 | 3 | -1 | 15642 | 6990 | 33897 | 58341 | 26102 | 0,03 | 5,23 |
| Acetyl-coenzyme A carboxylase carboxyl transferase subunit beta | 606,46 | ACCD | 2 | 10275 | -1 | -1 | 22261 | 27395 | 14992 | 0,01 | 6,29 |
| Phosphomethylpyrimidine synthase | 550,48 | A9H8N9 | 2 | 4017 | -1 | 4924 | 27687 | 36679 | 13266 | 0,04 | 8,68 |
| 2-oxoglutarate dehydrogenase E1 component | 484,38 | A9HFG6 | 3 | 3132 | -1 | -1 | 34093 | 33410 | 9295 | 0,05 | 24,50 |
| Multidrug resistance protein A | 843,70 | A9H3B5 | 2 | 19702 | 13961 | 15624 | -1 | -1 | 12058 | 0,04 | 0,24 |
| Putative GcrA cell cycle regulator | 1309,88 | A9H9B8 | 2 | 17970 | 17088 | 30873 | 10146 | -1 | 9661 | 0,03 | 0,30 |
| Cold shock protein (Fragment) | 38029,39 | A5YJ14 | 3 | 206394 | 210724 | 344955 | 134666 | 122269 | -1 | 0,03 | 0,34 |
| Methylmalonate-semialdehyde dehydrogenase | 720,52 | A9H5B4 | 3 | 33446 | 35361 | 7114 | -1 | 11320 | 8162 | 0,08 | 0,26 |
| Putative conjugal transfer | 498,34 | A9HSY7 | 2 | 28127 | 28110 | 24712 | 13824 | -1 | 20313 | 0,06 | 0,42 |
| Acetolactate synthase, small subunit | 1098,25 | A9GZJ1 | 2 | 27577 | 26160 | 29719 | 20455 | -1 | 22377 | 0,10 | 0,51 |
| Conserved protein | 341,36 | A9HL44 | 2 | 9438 | -1 | 6951 | 4518 | -1 | 7638 | 0,36 | 0,74 |
| Flagellar P-ring protein | 361,79 | A9HH48 | 2 | 17035 | 15944 | 15909 | 17558 | -1 | 20038 | 0,31 | 0,77 |
| ATPase associated with various cellular activities | 922,45 | A9H3C6 | 4 | 19375 | 17316 | 16001 | 17093 | -1 | 25516 | 0,35 | 0,81 |
| Uncharacterized protein | 1699,55 | A9HMM2 | 3 | 48080 | 46162 | -1 | 34367 | 20221 | 27576 | 0,41 | 0,87 |
| Alcohol dehydrogenase zinc-binding domain protein | 932,29 | A9HNN4 | 2 | -1 | -1 | 22827 | -1 | -1 | 19952 | 0,46 | 0,87 |
| Putative amidohydrolase | 667,85 | A9HMC5 | 2 | 28917 | 16935 | -1 | 16879 | -1 | 23539 | 0,44 | 0,88 |
| dTDP-4-dehydrorhamnose 3,5-epimerase | 698,74 | A9H3H9 | 2 | 9511 | 9347 | 15355 | 8591 | -1 | 22077 | 0,44 | 0,90 |
| Anthranilate synthase component 1 | 388,23 | A9HJ91 | 3 | -1 | -1 | 11963 | -1 | -1 | 10940 | 0,48 | 0,91 |
| Uncharacterized protein | 662,74 | A9HFW9 | 2 | 18166 | 18241 | -1 | 15611 | -1 | 17937 | 0,46 | 0,92 |
| Conserved protein | 3664,53 | A9H9B9 | 3 | 47465 | 38701 | 27456 | -1 | 21152 | 93730 | 0,49 | 1,01 |
| 3'(2'),5'-bisphosphate nucleotidase CysQ | 759,11 | A9H1J6 | 3 | 13041 | 13287 | 8573 | 11586 | 1 | 24020 | 0,49 | 1,02 |
| Endoribonuclease L-PSP | 4143,12 | A9HC24 | 4 | -1 | -1 | 95689 | -1 | -1 | 99557 | 0,49 | 1,04 |
| 1-(5-phosphoribosyl)-5- imidazole-4-carboxamide | 613,08 | A9GZX2 | 3 | 35860 | 29221 | -1 | 42313 | -1 | 26416 | 0,47 | 1,06 |
| Putative Antibiotic biosynthesis monooxygenase | 1420,20 | A9HP28 | 2 | 23867 | 52285 | -1 | 39979 | 40624 | -1 | 0,47 | 1,06 |
| Putative 2-nitropropane dioxygenase | 589,05 | A9HJ45 | 3 | 33461 | 34026 | -1 | 33564 | 23687 | 16977 | 0,43 | 1,10 |
| Mammalian cell entry related domain protein | 337,99 | A9H983 | 2 | -1 | 12369 | 17645 | 17697 | 12986 | 11705 | 0,26 | 1,41 |
| Glutamine amidotransferase of anthranilate synthase | 668,96 | A9HJ94 | 2 | -1 | -1 | 12193 | 6344 | -1 | 12049 | 0,36 | 1,51 |
| Phosphatidylserine decarboxylase proenzyme | 721,36 | PSD | 2 | 29068 | 31079 | -1 | 34843 | 39755 | 25355 | 0,16 | 1,66 |
| Universal stress protein | 557,72 | A9H0V0 | 2 | 36449 | 1 | 17141 | 35012 | 36338 | 27181 | 0,14 | 1,84 |
| Outer membrane protein (OprJ) | 611,21 | A9HA51 | 3 | 26281 | 23586 | -1 | 29329 | 51379 | 30878 | 0,07 | 2,24 |
| 3-hydroxyacyl-[acyl-carrier-protein] dehydratase | 348,16 | FABZ | 2 | 1 | 6308 | 8585 | 8686 | 7902 | 20272 | 0,11 | 2,47 |
| Transcriptional regulator, CarD family (GDI1850) | 816,34 | A9HIN7 | 3 | -1 | 6915 | 4314 | 15460 | -1 | 17646 | 0,16 | 2,95 |
| Fumarate hydratase class I (FumA) | 587,90 | A9HBG7 | 2 | 13447 | 11920 | -1 | 54265 | -1 | 20846 | 0,20 | 2,96 |
| *TIC - Total Ion Counts |  |  |  |  |  |  |  |  |  |  |  |
